# Illuminating the Black Box: A Perspective on Zeolite Crystallization in Inorganic Media

**DOI:** 10.1021/acs.accounts.3c00269

**Published:** 2023-08-11

**Authors:** Karel Asselman, Christine Kirschhock, Eric Breynaert

**Affiliations:** † Center for Surface Chemistry and Catalysis − Characterization and Application Team (COK-KAT), KU Leuven, 3001 Leuven, Belgium; ‡ NMR/X-ray Platform for Convergence Research (NMRCoRe), KU Leuven, 3001 Leuven, Belgium

## Abstract

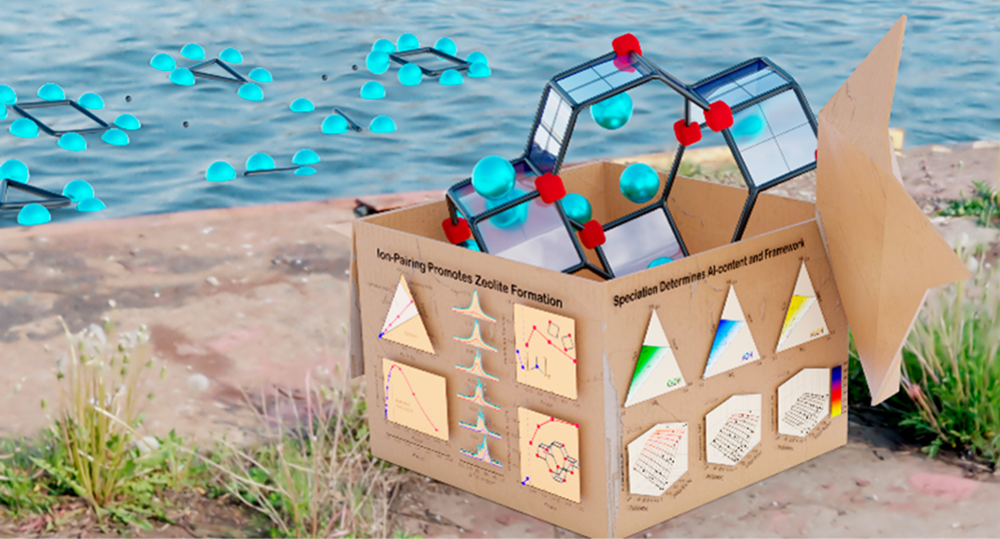

Since the discovery of synthetic zeolites in the 1940s and their implementation in major industrial processes involving adsorption, catalytic conversion, and ion exchange, material scientists have targeted the rational design of zeolites: controlling synthesis to crystallize zeolites with predetermined properties. Decades later, the fundamentals of zeolite synthesis remain largely obscured in a black box, rendering rational design elusive. A major prerequisite to rational zeolite design is to fully understand, and control, the elementary processes governing zeolite nucleation, growth, and stability. The molecular-level investigation of these processes has been severely hindered by the complex multiphasic media in which aluminosilicate zeolites are typically synthesized. This Account documents our recent progress in crystallizing zeolites from synthesis media based on hydrated silicate ionic liquids (HSIL), a synthesis approach facilitating the evaluation of the individual impacts of synthesis parameters such as cation type, water content, and alkalinity on zeolite nucleation, growth, and phase selection. HSIL-based synthesis allows straightforward elucidation of the relationship between the characteristics of the synthesis medium and the properties and structure of the crystalline product. This assists in deriving new insights in zeolite crystallization in an inorganic aluminosilicate system, thus improving the conceptual understanding of nucleation and growth in the context of inorganic zeolite synthesis in general. This Account describes in detail what hydrated silicate ionic liquids are, how they form, and how they assist in improving our understanding of zeolite genesis on a molecular level. It describes the development of ternary phase diagrams for inorganic aluminosilicate zeolites via a systematic screening of synthesis compositions. By evaluating obtained crystal structure properties such as framework composition, topology, and extraframework cation distributions, critical questions are dealt with: Which synthesis variables govern the aluminum content of crystallizing zeolites? How does the aluminum content in the framework determine the expression of different topologies? The crucial role of the alkali cation, taking center stage in all aspects of crystallization, phase selection, and, by extension, transformation is also discussed. New criteria and models for phase selection are proposed, assisting in overcoming the need for excessive trial and error in the development of future synthesis protocols.

Recent progress in the development of a toolbox enabling liquid-state characterization of these precursor media has been outlined, setting the stage for the routine monitoring of zeolite crystallization in real time. Current endeavors on and future needs for the in situ investigation of zeolite crystallization are highlighted. Finally, experimentally accessible parameters providing opportunities for modeling zeolite nucleation and growth are identified. Overall, this work provides a perspective toward future developments, identifying research areas ripe for investigation and discovery.

## Key References

- van TendelooL.; HaouasM.; MartensJ. A.; KirschhockC. E. A.; BreynaertE.; TaulelleF.Zeolite Synthesis in Hydrated Silicate Ionic Liquids. Faraday Discuss.2015, 179, 437–44910.1039/C4FD00234B25886652.^1^*Hypohydrated, liquid silicate solutions were introduced as zeolite precursors, functioning as ideal model systems for inorganic zeolite synthesis. Their versatility was explored by the variation of the alkali cation and water content, yielding various zeolite topologies*.
- AsselmanK.; PellensN.; ThijsB.; DoppelhammerN.; HaouasM.; TaulelleF.; MartensJ. A.; BreynaertE.; KirschhockC. E. A.Ion-Pairs in Aluminosilicate-Alkali Synthesis Liquids Determine Aluminium Content and Topology of Crystallizing Zeolites. Chem. Mater.2022, 34( (16), ), 7150–715810.1021/acs.chemmater.2c0077336032556PMC9404546.^2^*The development of ternary crystallization diagrams for HSIL-based synthesis mixtures revealed a direct, continuous relation between synthesis descriptors and the aluminum content of the resulting zeolite structure*.
- AsselmanK.; VandenabeeleD.; PellensN.; DoppelhammerN.; KirschhockC. E. A.; BreynaertE.Structural Aspects Affecting Phase Selection in Inorganic Zeolite Synthesis. Chem. Mater.2022, 34( (24), ), 11081–1109210.1021/acs.chemmater.2c0320436590702PMC9798827.^3^*The connection between framework aluminum content and topology was revealed via analysis of the cation sites and distributions in inorganic zeolites. A novel phase selection criterion for inorganic zeolite synthesis allows us to predict the synthesis outcome based on the maximization of the framework–cation interaction energy*.
- PellensN.; DoppelhammerN.; RadhakrishnanS.; AsselmanK.; Vinod ChandranC.; VandenabeeleD.; JakobyB.; MartensJ. A.; TaulelleF.; ReichelE. K.; BreyneartE.; KirschhockC. E. A.Nucleation of Porous Crystals from Ion-Paired Pre-Nucleation Clusters. Chem. Mater.2022, 34( (16), ), 7139–714910.1021/acs.chemmater.2c0041836032557PMC9404542.^4^*Multidiagnostic analysis of the molecular speciation in HSIL-based synthesis liquids identified the prevalence of ion association between soluble (alumino)silicate oligomers and alkali cations and the importance of this interaction for zeolite nucleation*.

## Introduction

Natural zeolites had been known for centuries, but only in 1948 was Barrer able to crystallize the first synthetic zeolite.^5^ Following this achievement, the zeolite community focused on the discovery and development of new zeolites and zeolite applications. Mechanistic analysis of hydrothermal zeolite crystallization from gels and sols received increasing interest in the 1990s and early 2000s, summarized in an extensive review by Cundy and Cox in 2005.^6^ The typical inorganic zeolite synthesis occurs through the hydrothermal conversion of aluminosilicate gels, involving complex multiphasic, highly alkaline media combined with high temperatures and pressures. These conditions have severely hindered molecular-level investigations of zeolite crystallization. In particular, the heterogeneous nature, encountered in sol–gel syntheses, poses problems. As a result, studies primarily relied on ex situ analysis of kinetic aspects of zeolite crystallization. Despite these experimental hurdles, several solution-mediated growth models consistent with experimental observations emerged over the years. At present, the literature suggests that zeolite nucleation and growth can occur from molecular aluminosilicate species in the precursor liquid, at the solid–liquid interphase of precursor aggregates, or internally in gel particles.^6,7^ Regardless of the phase where nucleation and growth occur, water is almost always present in the synthesis, either as a solvent or as a reactant.

Renewed activity in molecular investigations of zeolite growth media was sparked by the discovery of high-silica MFI, silicalite-1, and zeolite beta “clear solution” syntheses.^8−11^ These typically depart from tetraethylorthosilicate (TEOS) as the silicon source and quaternary ammonium organic structure-directing agents (OSDA). While the usually dilute “clear solution” media appear clear to the eye, they are suspensions of nanoaggregates, suggested as zeolite precursors.^12^ Compared to traditional aluminosilicate gel syntheses, these “clear solutions” are compatible with many characterization techniques and became prototype systems for investigation. However, their colloidal nature still encumbers the analysis of dissolved and aggregated species and their respective roles. Furthermore, these systems are highly dilute, as compared to typical inorganic aluminosilicate sol–gel systems, where an evolving gel network is in chemical exchange with a liquid phase. Until now, “clear solution” media are also restricted to a few topologies, limiting the options to draw general conclusions.

While the introduction of OSDAs revolutionized zeolite science, there is a general consensus that economic and sustainable zeolite synthesis should occur from purely inorganic media. At present, however, inorganically produced zeolites have high framework aluminum contents, with Si/Al ratios typically ranging between 1 and 5. While these highly ionic aluminosilicate materials find increasing attention for bulk industrial processes such as ion exchange, adsorption, and gas separation, most catalytic applications rely on zeolites with a higher silicon content. Inorganic synthesis of silicon-rich zeolites, consequently, remains an active area of research. Progress in this field critically depends on the development of new insights into the black box of inorganic zeolite formation, for which many key aspects are still elusive and under debate. The structure-directing role of the inorganic cation,^13,14^ the relative importance of kinetics and thermodynamics, and the impact of water,^15^ among others, are still disputed. Simple inorganic synthesis media, representative of general inorganic synthesis and compatible with molecular-level investigation, are therefore essential to unraveling the intricacies of zeolite formation.

## Hydrated Silicate Ionic Liquids

**Figure 1:**
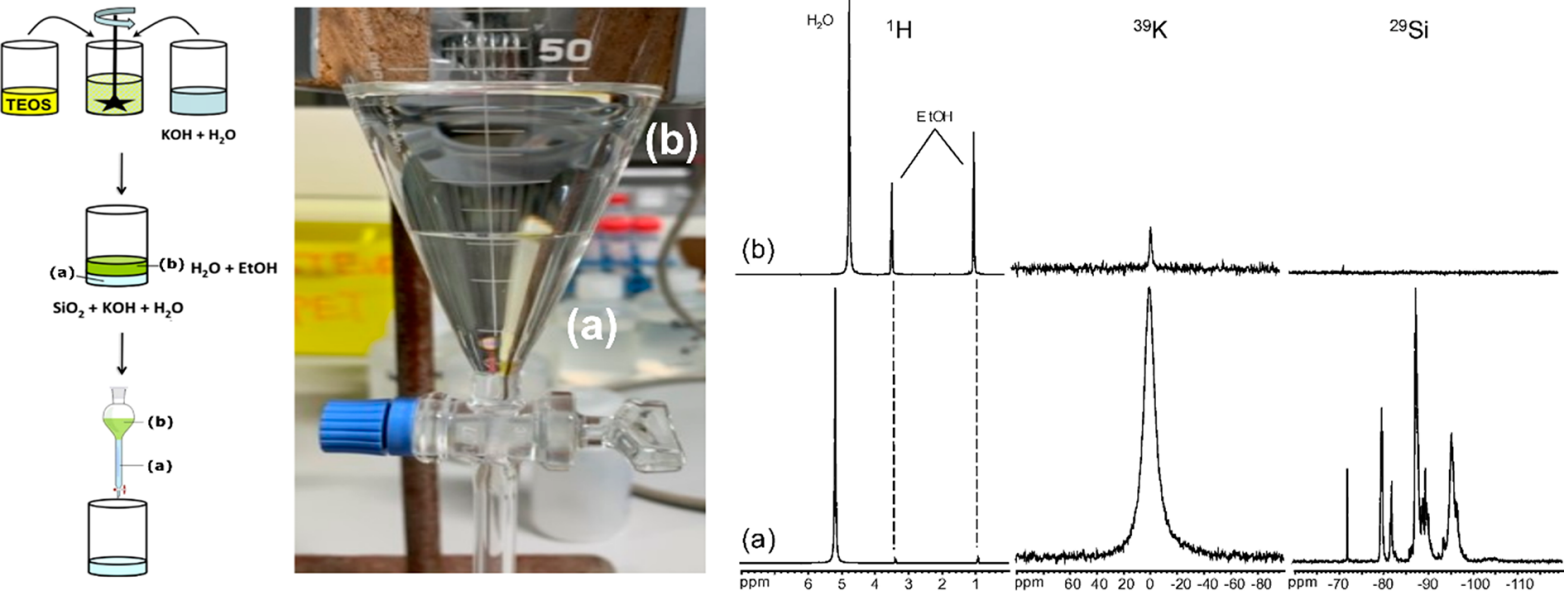
(Left) Preparation of a hydrated silicate ionic liquid. A mixture with composition 1 TEOS:1 KOH:20 H_2_O spontaneously phase separates upon TEOS hydrolysis in an upper water–ethanol (b) and bottom potassium HSIL phase (a) with nominal composition 1 Si(OH)_4_:1 KOH:3.6 H_2_O. (Center) Image of phase separation in a separation funnel. (Right) ^1^H, ^39^K, and ^29^Si liquid-state NMR characterization of upper water–ethanol and bottom HSIL phases. Adapted from ref (16) with permission. Copyright 2014 Elsevier.

Discovered by Francis Taulelle and co-workers in 2014, hydrated silicate ionic liquids (HSILs) were identified as potential new model systems for studying inorganic zeolite formation.^1^ HSILs form through spontaneous liquid–liquid demixing in TEOS–H_2_O–MOH (M = Na^+^, K^+^, Rb^+^, Cs^+^) systems.^1,16^ Upon hydrolysis of TEOS, spontaneous coacervation separates a water–ethanol phase from the dense HSIL phase (Figure 1), a common observation in the presence of highly soluble inorganic electrolytes.^17^ The dense HSIL phase contains nominally all silicate, alkali hydroxide, and residual hydration water (2–4 H_2_O molecules per cation). HSILs are predominantly ionic in nature, optically transparent, monophasic, and do not contain aggregates or colloids.^1,16^ Rather than being a solvent, water acts as a reactant involved in hydrogen bonding and continuous hydrolysis/condensation processes with silica-oligomers. Likewise, a large fraction of hydroxide ions are consumed in silicate deprotonation. Cation charge is mostly compensated for by interaction with deprotonated silanols and a few residual hydroxide ions in a hydrogen-bonded network. The local environment of the cations is thus dominated by interaction with protonated and deprotonated silanols and contains only a few water molecules. In combination, this results in a dynamic network of ion-paired species in the HSIL phase.^4,18^

Successful HSIL formation occurs within a well-defined range of molar ratios in the TEOS–H_2_O–MOH ternary diagram.^16^ Too much water or TEOS results in the failure of phase separation or the precipitation of a gel. Successful coacervation depends on the minimization of enthalpy by maximized Coulombic interaction between silicate anions and the alkali cations, achieved by high degrees of silicate deprotonation (*n*(OH^–^)/*n*(TEOS) ≥ 1). Water, usually needed for hydration, is released into the ethanolic/aqueous phase with high configurational entropy. In this way, the free energy of the two-phase system is minimized. The ^29^Si, ^1^H, and ^39^K NMR spectra of a native K-HSIL phase with composition 1 SiO_2_:1 KOH:3.6 H_2_O and the separated ethanol–water phase are shown in Figure 1. The composition of the native HSIL can be adjusted by the addition of extra alkali hydroxide or water to reach a desired synthesis stoichiometry.

**Figure 2:**
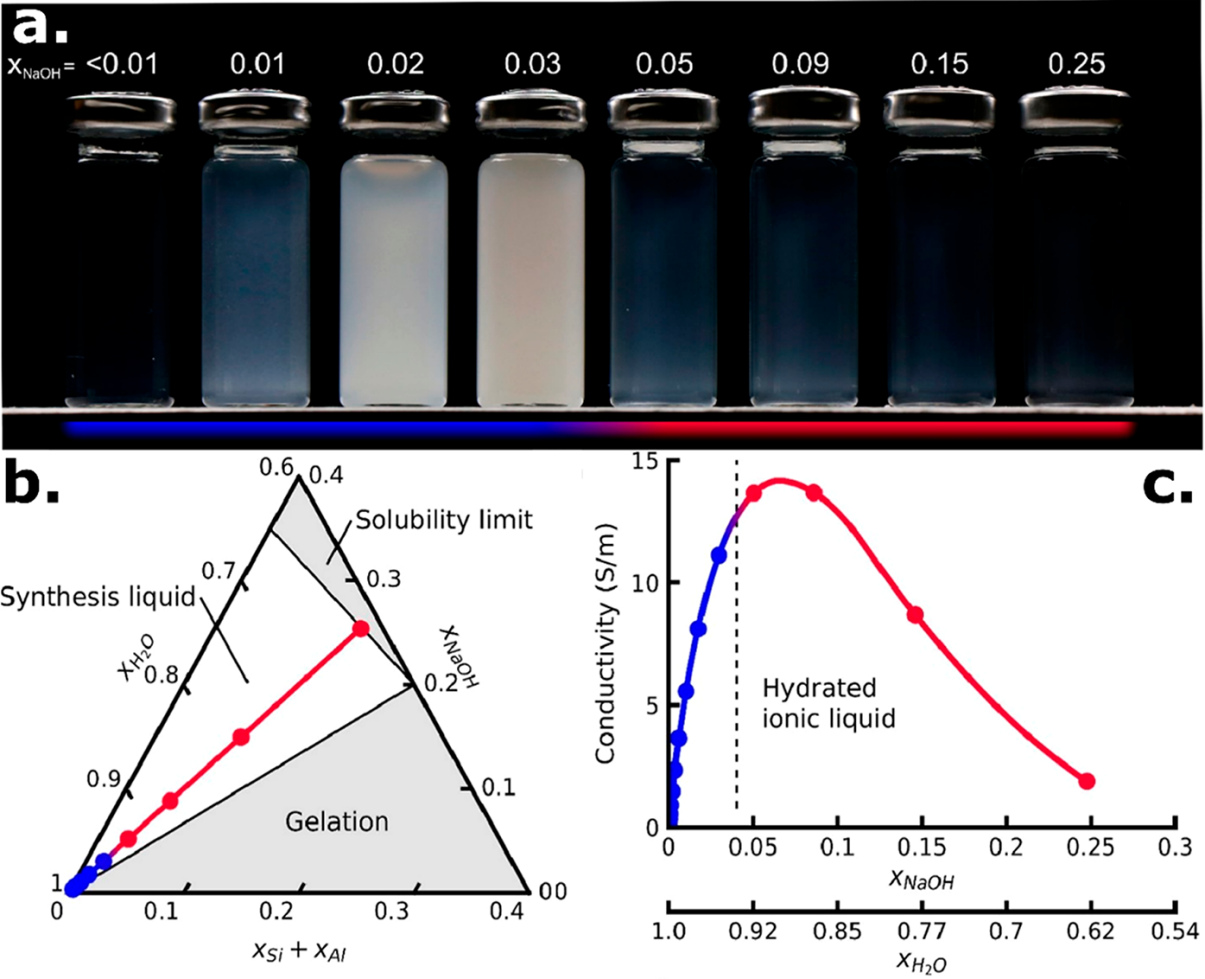
Ion association and aluminosilicate aggregation in Na-HSIL synthesis media as a function of water content, with molar composition 0.5 Si(OH)_4_:0.028 Al(OH)_3_:1 NaOH:*z* H_2_O. (a) In the ionic liquid domain (red), ion association between aluminosilicate oligomers and alkali cations stabilizes the solution, resulting in particulate-free, homogeneous liquids. Dilution results in increased cation hydration and separation of ion pairs, resulting in aluminosilicate aggregation and the formation of a colloidal suspension. (b) Stoichiometry of liquid samples indicated in the ternary diagram representation, with the transition from an ion-stabilized liquid (red) to a colloidal suspension (blue). Compositional regions resulting in spontaneous gelation and the solubility limit due to insufficient water are indicated. (c) Conductivity measurement of corresponding liquids. The maximum in the conductivity curve corresponds to the transition from a homogeneous liquid to a colloidal suspension. Left of the maximum, the conductivity decreased through dilution, while on the right, conductivity decreases due to ion association of alkali and (alumino)silicate. Adapted from ref (4) with permission. Copyright 2022 American Chemical Society.

Functioning as a silicon source for zeolite synthesis, HSIL-based precursor liquids are prepared via the addition of aluminate. Isomorphic substitution of Si by Al results in the spontaneous formation of aluminosilicate oligomers. The solubility of aluminate in these mixtures is higher than in aqueous media due to their high ionicity but still quite low. Therefore, only small concentrations of aluminate ([Al]_critical_) can be added before aggregates appear as aluminum-rich colloids. While this does not strongly affect the synthesis result, the presence of aggregate phases impedes the study of nucleation and crystal growth. Al solubility depends on the molar composition of the HSIL and the type of cation. The approximate requirements for (initially) homogeneous precursor liquids are a combination of low aluminate fractions (Si/Al > 20), sufficient alkalinity ([Si(OH)_4_]/[OH^–^] ≤ 0.5), and severe water restriction ([H_2_O]/[MOH]< ± 10) (Figure 2).^2,4^ While purely siliceous HSIL is stable indefinitely, aluminate addition yields metastable liquids. While these Al-doped HSILs also typically resist precipitation at room temperature, long-term incubation at room temperature can result in the formation of crystalline zeolite phases (Figure S1). Heating Al-doped HSIL results in rapid zeolite formation.

## Homogeneous Precursor Liquids Illuminate the Black Box of Zeolite Crystallization

Progress toward rational zeolite synthesis requires an accurate observation and a fundamental description of molecular and macroscopic properties of liquid and solid phases present during nucleation and growth. This can be achieved through molecular investigation of the liquid- and solid-state chemistry of synthesis mixtures before and after crystallization or, preferably, in situ during crystallization. In the liquid state, this includes a description of the chemical potential, equilibrium distribution, and speciation of (alumino)silicate oligomers and their ion association with alkali cations. In the solid state, the stability of the as-synthesized zeolite must be linked to the synthesis liquid in which it preferentially forms. This includes accounting for zeolite topology, Al content and distribution, cation type, and water content. Under equilibrium conditions, liquid- and solid-state speciation must ultimately be related through macroscopic descriptors, such as solubility products. Such descriptors have remained elusive for zeolites due to their incongruent dissolution and the complex multiphasic nature of the precursor mixtures.

Hydrated silicate ionic liquids present an ideal model system for the molecular study of zeolite crystallization because chemically, HSIL-based liquids are closely related to the liquid phase encountered in traditional sol–gel systems: they have a simple physiochemical nature and oligomeric speciation, containing no extra reagents. HSIL-based media are compositionally versatile. Batch alkalinity can vary as 0 < [SiO_2_]/[MOH] ≤ 1. Water and aluminate contents can be varied incrementally. An examination of HSIL-based synthesis in the gel-free regime holds potential to push a fundamental understanding of zeolite growth processes for the following reasons:

i. Accessible by a variety of diagnostics, HSILs allow the acquisition of molecular insights into the initial steps of zeolite formation. Low synthesis temperatures and relatively short crystallization times enable in situ studies to be compatible with many diagnostics.
ii. A fully dissolved, homogeneous precursor liquid eliminates complex variables, such as gel aging and reagent source or mixing conditions. It minimizes the phenomenon of “false” environments.^19^ Gels may contain concentration gradients with local volumes of unknown chemical composition that can be drastically different from the global batch composition, complicating the relation between overall batch stoichiometry and the local chemical composition of the environment wherein zeolites nucleate.
iii. Crystalline yield is governed by the amount of added aluminate. Low solid yield resulting from restricted aluminate content in homogeneous HSIL ensures minimal evolution of the chemical potential of other solutes during crystallization. Crucial variables such as pH evolve to a minor extent, reducing the modulation of the chemical environment as a function of synthesis time, facilitating chemical modeling.

In what follows, we present insights derived from the study of zeolite formation in HSIL-based precursor liquids. These insights are contextualized within the broader literature, providing a general perspective on aspects governing inorganic zeolite synthesis.

## Connecting the Chemical Speciation in Liquid and Solid Phases

### Solubility of a Zeolite in Its Synthesis Medium Governs the Framework Si/Al Ratio

The thermodynamic description of solubility is notoriously hard to define for complex materials with incongruent dissolution such as zeolites. The concept of solubility is nevertheless essential to understanding the crystallization, dissolution, and phase behavior of any mineral. The solubility product reflects the relative tendency of mineral constituents to either be incorporated into the crystalline product or remain dissolved and defines the thermodynamic stability of a zeolite in its growth medium,.^13,20^ It drives the key processes of zeolite crystallization and dissolution in a specific liquid. This is relevant both with respect to synthesis strategies involving seeding and interzeolite conversion as well as to long-term applications of zeolites as an ion exchanger, for example, in environmental remediation or the disposal of radioactive waste.^21−26^

**Figure 3:**
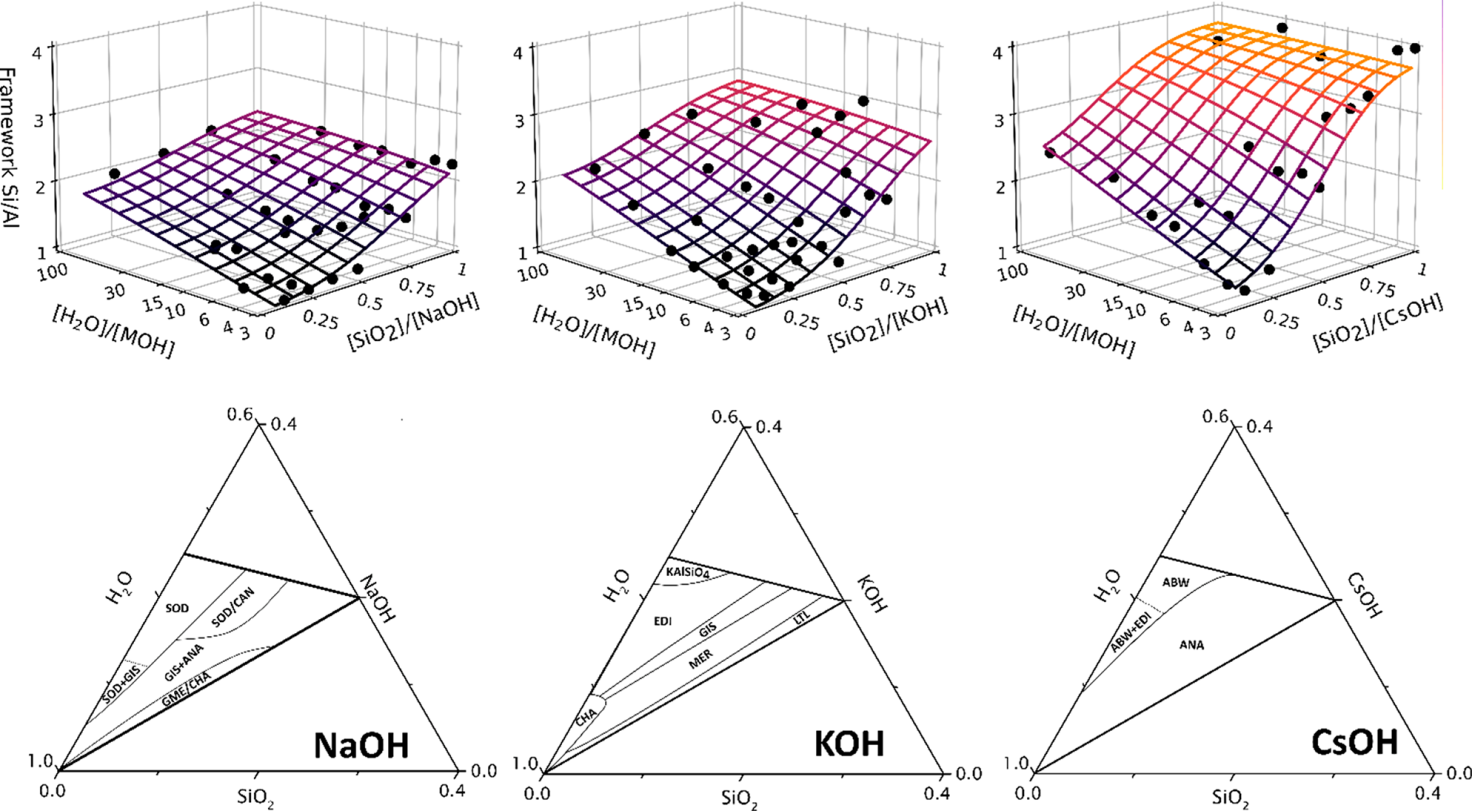
(Top) Evolution of the framework Si/Al ratio as a function of batch stoichiometry in the 0.5 SiO_2_:0.025 Al(OH)_3_:*x* MOH:*z* H_2_O system (M = Na, K, Cs), revealing a continuous correlation among the batch alkalinity, dilution, and cation type on the Si/Al ratio of crystallizing zeolites. (Bottom) Phase selection in the same synthesis data in the ternary diagram representation, after crystallization at 90 °C for 7 days. Framework variation in the Si/Al ratio with batch stoichiometry shows no discontinuities, but ternary crystallization diagrams show sharp phase boundaries between topologies. Adapted from ref (2) with permission. Copyright 2022 American Chemical Society.

The solubility of any mineral is governed by chemical speciation and the concentration of its constituents in a liquid. Contrary to popular discourse, zeolites are no exception. At equilibrium, the composition of the crystalline (alumino)silicate lattice together with its extraframework species determines the composition of the liquid phase and vice versa. Consequently, the relative stability of different zeolite phases in contact with a supernatant is defined by the composition of the liquid medium.^27^ This solid–liquid equilibrium, specifically the type and solubility of aluminosilicate oligomers in the HSIL, is ultimately tied to the framework Si/Al ratio of the final, thermodynamically preferred zeolite product. This was confirmed by the continuous relation between the molar composition of homogeneous HSIL-based syntheses and the Si/Al ratio of the resulting zeolites (Figure 3).^2,28^ A quantitative description of this relation will require the explicit determination of the solubility products for aluminosilicate zeolites in their respective growth media. HSIL-based zeolite synthesis facilitates this since the total solid yield is controlled by the amount of aluminum introduced in the synthesis. At low yields, the release of water and alkali-hydroxide during crystallization is limited, and the physiochemical properties of the synthesis liquid can be assumed to be quasi-constant. By now, a qualitative description of the impact of synthesis liquid compositional variables affecting the zeolite Si/Al ratio can be outlined:

i. Batch alkalinity. The batch alkalinity strongly affects the framework Si/Al ratio. Increasing batch alkalinity increases silicate deprotonation, subsequently decreasing the oligomerization and average connectivity ⟨*n*⟩ of Al to Si centers.^28^ These changes are reflected in the solid, resulting in a decrease in the framework Si/Al ratio.
ii. Cation type*.* With increasing size, monovalent cations display a reduction in charge density and increased polarizability, corresponding to a lower hydration energy. As a direct result, NMR investigations demonstrated that larger cations show an increased preference for direct coordination to (alumino)silicate oligomers, reducing the fraction of water molecules in their coordination shell.^29,30^ Furthermore, large alkali cations selectively coordinate to larger oligomers.^30^ This preference impacts the liquid speciation by shifting the oligomer distribution toward larger oligomers typically with increased Si/Al ratios, which is reflected in the resulting aluminum content of the zeolite framework (Figure 3).^30,31^
iii. Dilution. The role of water is complex and intricately connected to other synthesis parameters, indirectly impacting both effects of batch alkalinity and cation type. Overall, the water content strongly affects the framework aluminum content (Figure 3). Increased dilution decreases the effective alkalinity in the liquid and reduces the overall charge density in the system, resulting in increasing Si/Al values of the products. Furthermore, water competes with (alumino)-silicate oligomers as a coordination partner for the cations. Dilution therefore modulates the impact of cation type and batch alkalinity, also affecting the obtained product.

### Framework Aluminum Content Defines the Obtained Zeolite Topologies

**Figure 4:**
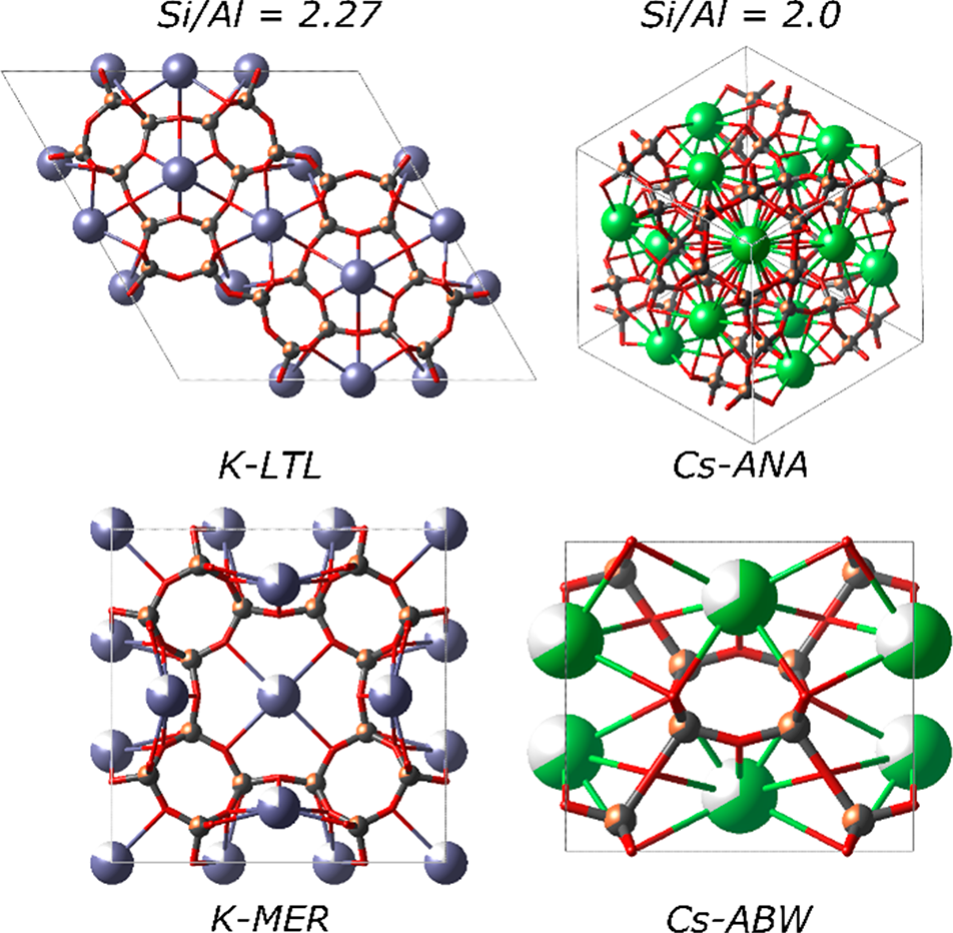
Aluminum content of the framework determines the selection of the zeolite topology. For given Si/Al ratio, the fractional occupancy of cation sites differs between topologies. Experimental zeolite products prefer the topology with the fewest vacant cation positions. Illustrated is the difference in cation occupancy between two common K- (violet) and Cs-bearing (green) frameworks. The cation occupancy is indicated with fractional coloring. Adapted from ref (3) with permission. Copyright 2022 American Chemical Society.

**Figure 5:**
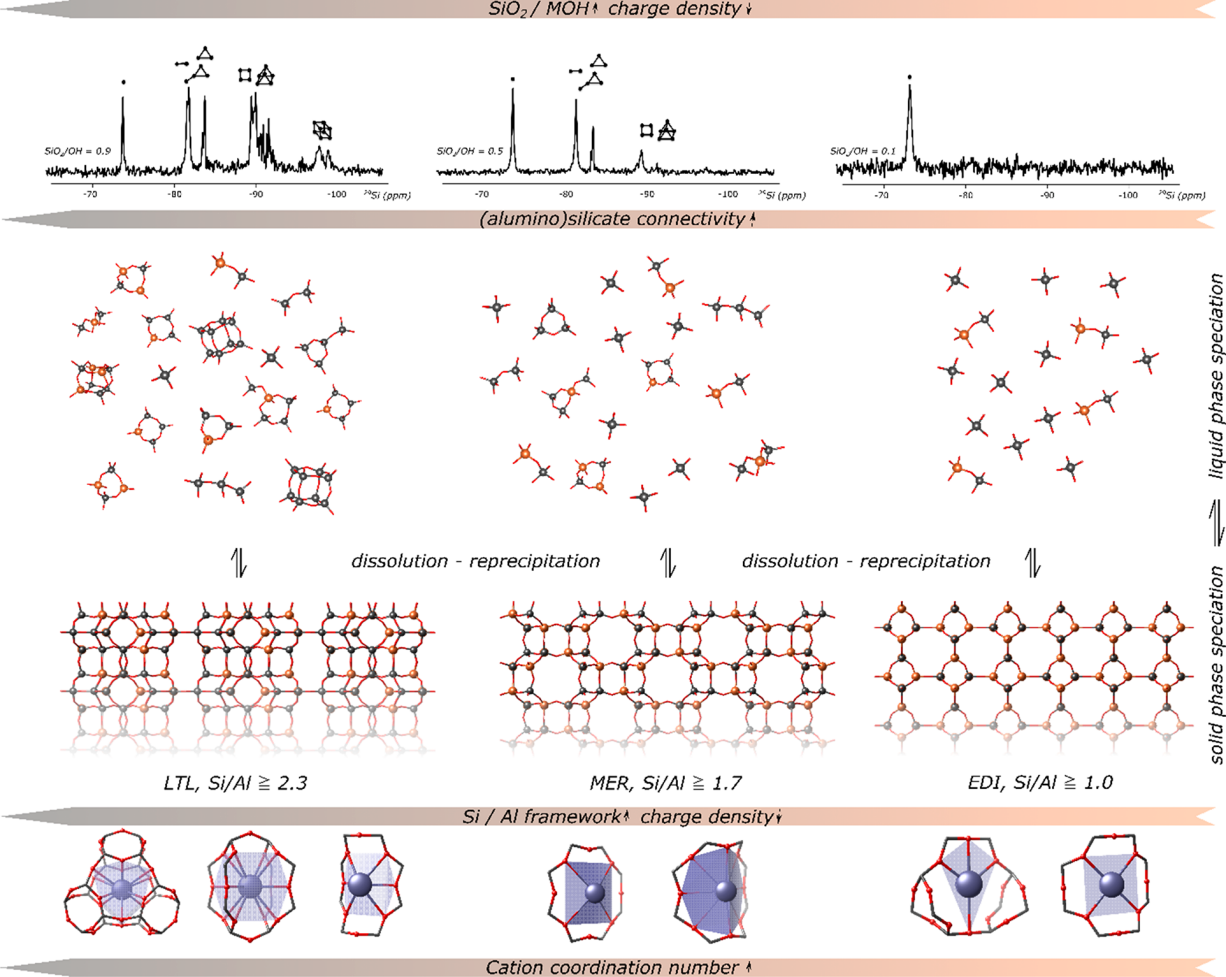
Generalized scheme for phase selection in inorganic media as a function of the liquid composition, illustrated for variable batch alkalinity [SiO_2_]/[MOH]. The aluminum content and topology can be rationalized via thermodynamic considerations such as solubility and crystal energy. The composition of the synthesis batch dictates the oligomerization in the liquid and governs the final framework Si/Al ratio. Selection of the topology offering maximized cation-framework stabilization, illustrated here for some potassium zeolites, is a consequence hereof.

In contrast to the continuous relation between the batch composition and framework Si/Al ratio, different zeolite topologies typically crystallize in sharply delineated regions in the crystallization diagrams (Figure 3).^2^ By systematically varying the composition of syntheses mixtures, the stability regions of crystallizing topologies were identified (Figure 1). While the Si/Al ratio of the framework is entirely defined by the composition of the synthesis liquid through solubility, phase selection apparently depends on relative stabilities of zeolite topologies at the given Al content in the framework. The concept of topological framework density is often used as a proxy for the relative stability of zeolites, even though this relation is valid only for siliceous frameworks after pores are already emptied.^32^ During zeolite synthesis, the formed products are not porous but contain extra-framework species such as cations and hydration water. In aluminosilicates, the framework stoichiometry is much more decisive for the crystal energy than slight changes in the topological framework density.^33^ It is critical to appreciate that extra-framework cations are not merely bystanders in the zeolite structures required for charge compensation. They occupy specific sites in each zeolite framework, with maximum coordination to framework oxygen, and codetermine its stability. For a given aluminum content and cation type, the topology with optimal stabilization of framework and extra-framework constituents is the thermodynamically preferred product, implying the minimization of cation vacancies.^3^ Evaluating zeolite topologies and their typical cation distributions allowed the postulation of a coherent selection criterion for zeolite phases crystallizing from inorganic media.^3^ For a specific framework Si/Al ratio, the topology with the highest fractional occupancy of its cation sites is preferred. The number of viable sites for a given cation in a topology, defined by its structure, strictly limits the energetically feasible aluminum content for the product, defining a cation-dependent “minimal” Si/Al ratio for each zeolite framework (Figures 4 and 5).^3^ When there are fewer aluminum sites than viable cation positions, i.e., the framework Si/Al is higher than the theoretical minimum, some cation sites must be vacant to preserve charge neutrality. If another topology is able to accommodate the same number of cations but with fewer vacancies, then this topology will take thermodynamic precedence. The phase selection criterion reflects the tendency of a zeolite crystal to optimize mutual interactions between the framework and extra-framework species and explains the strict demarcation and sudden phase transitions encountered in crystallization diagrams. This concept demystifies why a single alkali cation may template so many different framework topologies in similar synthesis media.

### Hydration Free Energy Favors Porosity

In addition to the extra-framework cations, water inherently codetermines the crystal energy. From an enthalpy perspective, porous, hydrated frameworks are usually favored over anhydrous aluminosilicates. At low temperatures, the enthalpy of hydration readily compensates for the metastability of the dehydrated porous framework, as evidenced by calorimetric studies.^33−35^ The synthesis temperature exerts the strongest influence on the water content and the corresponding porosity. Identical precursor liquids often crystallize multiple zeolite topologies or anhydrous aluminosilicates by varying the synthesis temperature. For instance, a precursor mixture with composition 0.5 Si(OH)_4_:0.025 Al(OH)_3_:5 KOH:50 H_2_O yields K-BPH, K-EDI, and megakalsilite, a feldspathoid, at synthesis temperatures of 50, 100, and 170 °C, respectively. These phases have identical anhydrous compositions of KAlSiO_4_ but differ in the amount of hydration water in their pore systems. Similar phase behavior with temperature is ubiquitously observed in inorganic zeolite synthesis.^36,37^ Comparing the solid phases, they typically exhibit similar framework compositions and display (quasi)identical cation occupancy numbers with decreasing pore volume and water content. Low synthesis temperatures favor porous, hydrated topologies due to favorable hydration enthalpy. At increased synthesis temperatures, the unfavorable configurational entropy of confined water^38^ results in denser structures with fewer water molecules and eventually anhydrous feldspathoids.^34^ We recently demonstrated that, for similar reasons, lowering the water activity in the liquid through extreme water restriction favors dense, nonporous frameworks.^15^ Likewise, the variation of hydration energy versus framework interaction for the different cations determines the water content in the zeolite product and the resulting topology. For this reason, sodium zeolites typically contain more water than cesium-derived products. This concept also suggests why temperature-induced phase transformations with gradual changes in confined hydration water are more frequently observed in Na-containing synthesis media as compared to media predominantly containing K and Cs cations.^36,37^

## In Situ Characterization of Zeolite Crystallization in HSIL Synthesis Media: A Pathway toward a Molecular Description of Zeolite Crystallization

For the full characterization of zeolite nucleation and growth, a combination of methods monitoring liquid and solid fractions in all aspects is necessary. In the following text, a promising combination of diagnostics will be listed. On the molecular level, NMR, probing for specific environments of elements, offers ideal opportunities to detect the removal of specific molecular components from solution as well as to detect density fluctuations generating larger species with a lifetime in the NMR observable range. On the particle level, X-ray scattering and diffraction readily yield the particle size and crystalline structure. For now, since NMR and X-ray scattering cannot be combined into a single multidiagnostic measurement, an independent reporter informing about the progress of aluminosilicate condensation is necessary to enable reliably combining results of the different methods. For this purpose, moving electrode electrochemical impedance spectroscopy has been developed. Combined, these diagnostics provide molecular-level access to all events occurring in the sequence of nucleation and growth of crystalline zeolites. At present, multidiagnostic in situ cells enabling combined MEEIS and XRD/SAXS as well as combined MEEIS and NMR are under development. This will facilitate the coregistration of the results obtained from multiple diagnostics for the same synthesis. In what follows, the information that can be obtained by the separate diagnostics in HSIL-based zeolite synthesis has been outlined.

### Probing the Chemical Environment of Different Elements in HSIL Precursor Mixtures: NMR Spectroscopy

As outlined above, in inorganic zeolite crystallization, aluminosilicate oligomers are the key species, and the solid yield directly correlates to the available aluminate. With its high sensitivity and short measurement times, liquid-state ^27^Al NMR is ideally suited to monitor the evolution of aluminosilicate during crystallization.^39^ Static and MAS NMR measurements are possible at temperatures of up to 90 °C and can be performed in a quantitative way,^40−42^ thus enabling in situ studies. Also here, homogeneous synthesis media such as HSILs excel for this purpose due to their relatively simple physiochemical nature.

**Figure 6:**
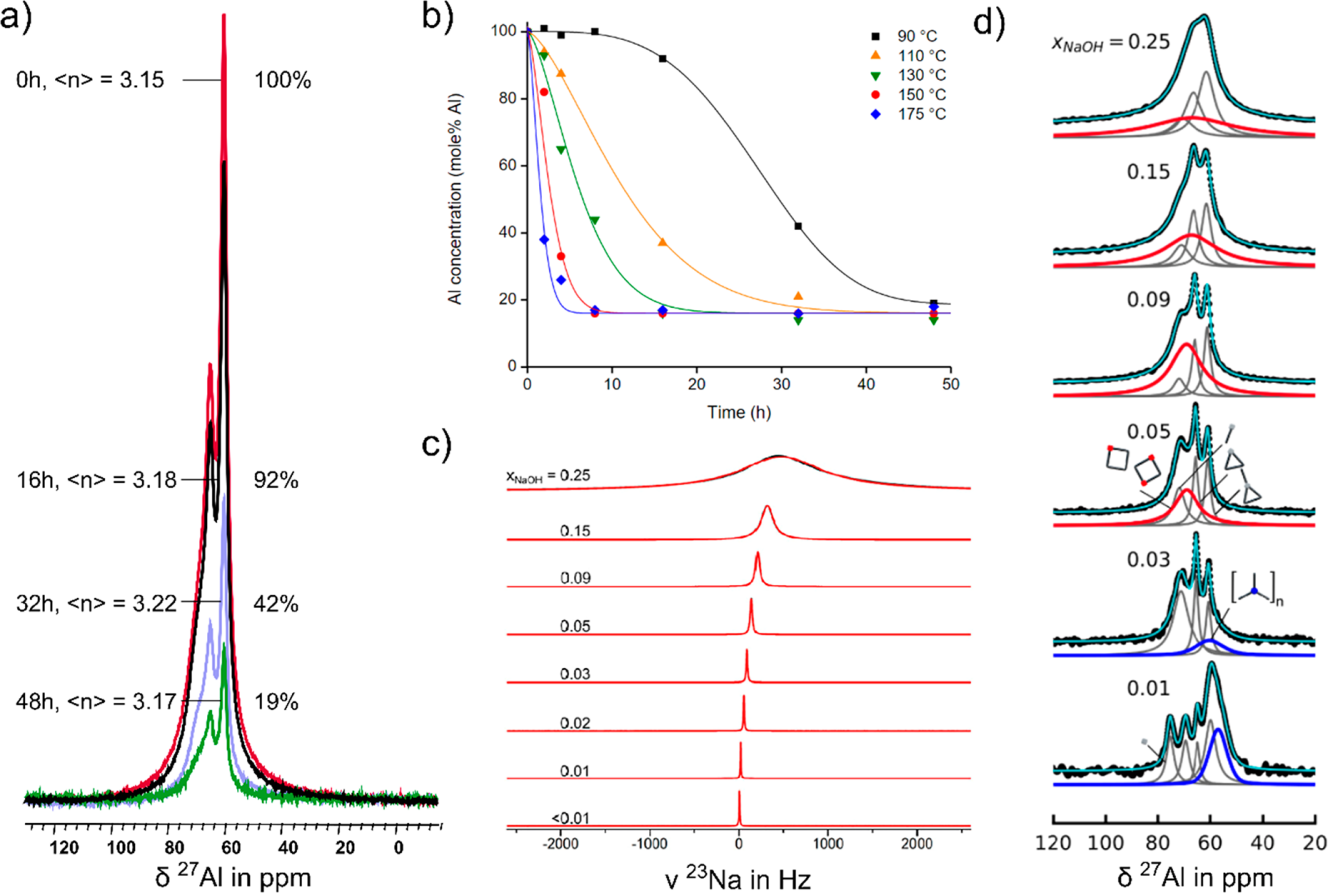
Starting from a homogeneous synthesis solution, liquid-state NMR allows the quantification and probing of the chemical environment of aluminosilicate oligomers and alkali cations. (a, b) Quantitative ^27^Al NMR analysis monitors the crystallization of zeolite W via the quantification of supernatant aluminate. No marked changes in Al connectivity ⟨*n*⟩ in the liquid was observed over time. Adapted from ref (44) with permission. Copyright 2019 Elsevier. (c, d) ^23^Na NMR allows the quantification of cation dynamics, hydration, and ion-pairing properties in solution as a function of batch composition 0.5 Si(OH)_4_:0.025 Al(OH)_3_:1 NaOH:*z* H_2_O via changes in chemical shift and line width analysis. For those synthesis compositions, the deconvolution of ^27^Al MAS NMR spectra allows the quantification of soluble aluminosilicate species responsible for crystallization (red resonances) and the detection of aggregated, amorphous aluminate (blue resonances). Adapted from ref (4) with permission. Copyright 2022 American Chemical Society.

For the sample series shown in Figure 6 (0.5 Si(OH)_4_:0.028 Al(OH)_3_:1 NaOH:*z* H_2_O), liquid ^27^Al MAS NMR measurements revealed a strong dependence of aluminate distribution on water content.^4^ A high spectral resolution allowed the assignment of specific aluminosilicate oligomers. Aluminosilicate four-rings, ion-paired to sodium cations, were identified as a potential species triggering the crystallization of GIS-type zeolites. For confirmation of this hypothesis, even greater resolution would be beneficial, for instance, by using higher magnetic fields or measurements at higher temperatures, exploiting motional narrowing.^43^

In a similar approach, following the crystallization of zeolite W, aluminate speciation and depletion were quantified via ^27^Al NMR, allowing an evaluation of the solubility limit of this zeolite upon equilibration in the crystallization medium.^44^ Spectra were acquired ex situ, periodically sampling the supernatants. While the kinetics of aluminate consumption by crystallization showed typical sigmoidal behavior, no marked change in aluminate connectivity in the supernatants was detected. This is in line with the assumption that low amounts of formed product do not significantly alter liquid speciation, especially considering the rapid interchange and equilibration of oligomers in the liquid. However, for further confirmation, in situ monitoring of aluminosilicate depletion via ^27^Al MAS NMR is desired. Such a study has the potential to identify, for the first time, the selective consumption of oligomeric growth units in real time.

Yielding much better resolved spectra, ^29^Si NMR in theory allows for the direct identification of soluble aluminosilicates, with chemical shifts well separated from their pure silicon analogues.^45^ However, low sensitivity and long measurement times restrict this approach for in situ monitoring.^4^ Most of those barriers can be resolved via the use of ^29^Si isotope-enriched reagent sources, but routine use is cost-prohibitive.

NMR has the unique ability to probe the coordination environment and dynamics of NMR active cations.^46−48^ By now it has become clear that the alkali cation affects all aspects of zeolite formation.^13,14^ It impacts the distribution of aluminosilicate species in the solution and selects the most stable framework topology. With ^23^Na, ^39^K, and ^133^Cs NMR, the degree of cations in the ion-paired state with oligomers versus hydrated cations in solution can be quantified (Figure 6).^4,15,29^ As expected, increasing dilution hydrolyses ion pairs, returning sodium to a fully hydrated state, resulting in the failure to form crystalline products at a low synthesis temperature of 60 °C. This prompts the hypothesis that ion pairing between alkali and aluminosilicate in the liquid is essential for successful nucleation and crystal growth.^4^ In more dilute mixtures, higher synthesis temperatures are required to favor the release of hydration water and provoke cation–oligomer pairing, allowing crystallization. Alternatively, phase separation into a dilute solution phase and a more concentrated gel phase can also provide conditions for nucleation.

### Detecting Nucleation and Crystal Growth: In Situ X-ray Scattering and Diffraction

**Figure 7:**
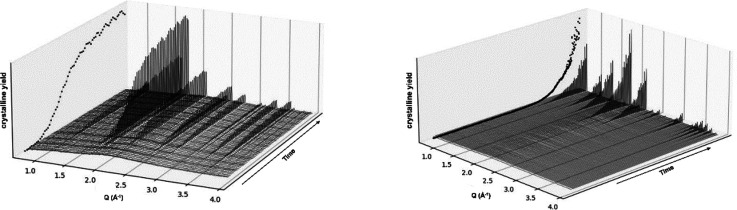
In situ monitoring of zeolite crystallization from HSIL-based media is possible by using X-ray scattering. Waterfall plots of WAXS patterns collected in situ during zeolite growth from HSIL for Cs-ANA (left) and Na-GIS (right) at 170 and 120 °C, respectively. Data collected at ESRF, Grenoble.^53,54^

X-ray diffraction is indispensable to the study of the structure of the crystalline product in detail, specifically focusing on the location of cations and hydration water.^18^ In the past, small-angle X-ray scattering (SAXS) was shown to be a versatile tool for detecting the presence and evolution of colloidal species in “clear solution” syntheses.^51,52^ It remains a promising method to verify if and how particulates with low internal ordering form prior to or during the nucleation of zeolites from HSIL. Combined in situ with X-ray diffraction, the appearance and topology of crystalline products can reveal the sequence of solids emerging from the liquid medium. Initial experiments demonstrated that in situ synchrotron X-ray scattering studies, monitoring zeolite crystallization in HSIL-based precursor liquids, is possible using a rudimentary, disposable setup of sealed quartz capillaries (Figure 7). These curves provide direct access not only to the crystalline yield but also to the molecular structure of the solids formed. The simple measurement geometry enables in situ X-ray scattering in combination with an additional technique such as Raman spectroscopy or with impedance spectroscopy.

### MEEIS: Detecting Ion Pairing and Hydroxide Release during Nucleation and Crystal Growth

**Figure 8:**
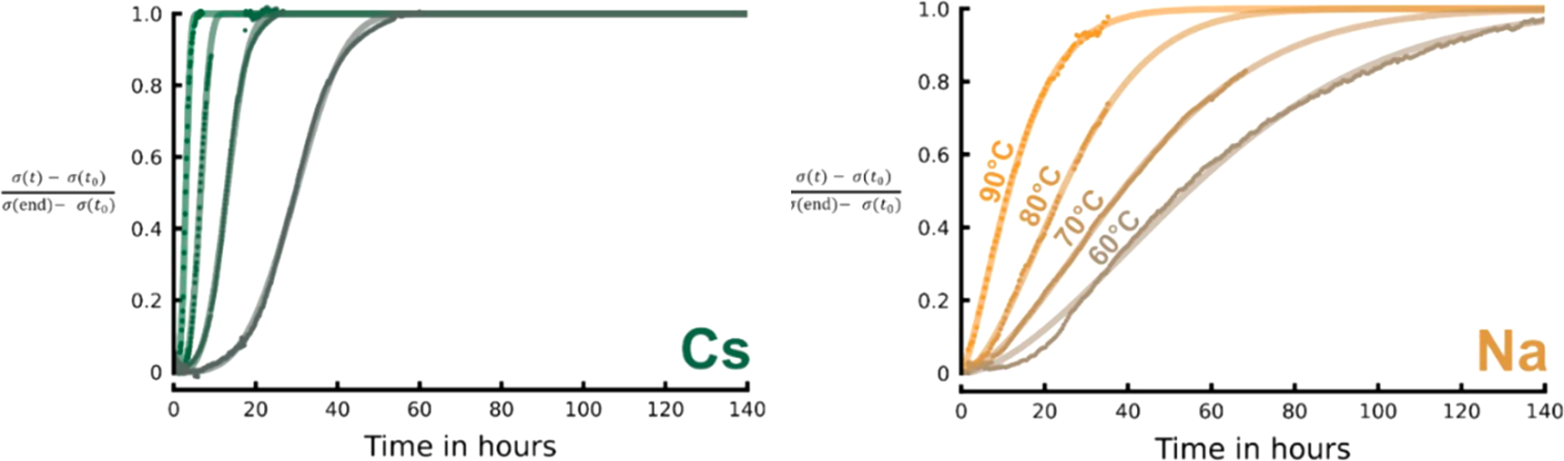
Crystallization curves for mixtures with molar composition 0.5 Si(OH)_4_:0.03 Al(OH)_3_:1 MOH:5 H_2_O (M = Na, Cs), measured via MEEIS as a function of temperature. Changes in bulk conductivity are a proxy for the solid yield. Reprinted from ref (55) with permission. Copyright 2021 Royal Society of Chemistry.

Inorganic crystallization media for zeolites are strong electrolytes. During crystallization, condensation reactions release hydroxide and previously ion-paired cations, thus increasing the bulk conductivity.^55^ A custom diagnostic for highly alkaline media, named moving electrode electrochemical impedance spectroscopy (MEEIS), was developed to allow in situ monitoring of minute conductivity changes in corrosive media at temperatures of up to 100 °C.^56^ MEEIS allows continuous monitoring for long time intervals (days to weeks), and artifacts arising from the electronics or sedimentation on the electrode are eliminated. This allows accurate measurements of the bulk conductivity in heterogeneous media.^57^ In HSIL, the evolution of bulk conductivity during the crystallization process is a cumulative, direct proxy for all condensation and hydrolysis processes occurring in the sample.^55^ Starting from homogeneous liquids, changes in conductivity therefore directly report the solid yield, facilitating a rapid collection of global crystallization curves. Thus far, conductivity data have been successfully measured to monitor the influence of synthesis temperature and the type of alkali cation in otherwise identical media of composition 0.5 Si(OH)_4_:0.03 Al(OH)_3_:1 MOH:5 H_2_O (M = Na, Cs) (Figure 8).^55^ Marked differences in crystallization profiles highlighted the importance of the alkali cation in the zeolite growth kinetics.

## HSIL Synthesis of Zeolites Paves the Way for Modeling

Zeolite synthesis in HSIL precursor media enables the in situ characterization of all components of a synthesis system. In situ NMR spectroscopy reveals the molecular-level speciation of the liquid phase throughout a synthesis. In situ X-ray scattering reveals the formation of particles potentially serving as nuclei, and their compositions can be derived by in situ NMR studies. In situ X-ray diffraction reveals the onset of crystalline growth and provides access to the long-range molecular-level structure of the growing crystals. Since in situ X-ray experiments are easily combined with in situ MEEIS and the NMR coil used during in situ NMR spectroscopy enables remote detection of the impedance of the sample inside the coil,^58^ in situ measurements obtained with different instruments can be synchronized and coregistered into a single data set using the bulk electrical properties of the synthesis liquid.

Even though a complete coregistered data set has not yet been produced, all separate in situ measurements have been performed and validated. Preliminary data sets have sparked discussion on potential kinetic models quantifying the observed zeolite nucleation and growth.^59^ First attempts, discussed in a recent Faraday Discussions on nucleation, describe zeolite growth as the combination of a reversible surface reaction followed by the transport of released hydroxide and surplus cations into the liquid.^55,60^ Future coregistered data sets will certainly allow for more detailed descriptions of growth kinetics. It is clear these data sets can provide experimental input for modeling strategies building on the concept of a chemical master equation, accounting for local processes at the interface between liquid and solid phases.^61^

## Conclusions

In controlled synthesis media, such as HSIL-based precursor mixtures, zeolite crystallization adheres to logical and established physiochemical and thermodynamic principles. Mechanistic insights derived from the study of these systems may be more widely representative of various inorganic zeolite synthesis systems. This is supported by the observation that zeolite products obtained from HSIL media are identical to those from classic sol–gel synthesis in terms of topology, composition, and morphology. Rather, HSIL-based synthesis minimizes complexity in the parameter space, such as gel aging, solid–liquid gel equilibria, and mixing conditions. Monophasic systems are less subject to complications resulting from compositional heterogeneity, false environments, etc. This dramatically reduces the risk of generating data sets influenced by unintended kinetic barriers or inhomogeneous crystallization environments, thus enhancing reproducibility.

This perspective summarized our view on zeolite crystallization and phase selection, gathered mostly from our own experiments but applicable in the wider context of zeolite synthesis. Phase selection was shown to be driven by the cation framework and, by extension, cation hydration energetics. Future investigations will further strengthen or challenge these concepts. To assist such evolution, the potential of HSIL in the molecular investigation of the precursor mixtures and in situ multidiagnostic monitoring of crystallization was outlined. Further exploiting the simple nature of these systems to investigate and revisit crystallization in aluminosilicate systems will assist in answering unresolved fundamental questions and push the frontier of the scientific description of the crystallization of complex materials.

## Supporting Information Available

XRD patterns of zeolite phases crystallized from HSIL media at room temperature after 6 months of incubation under stationary conditions (PDF)

## Dedication

This Account honors the memory of our esteemed friend and colleague, Prof. Francis Taulelle, who pioneered the investigation of hydrated silicate ionic liquids. Regrettably, his passing came before this groundbreaking discovery could fully flourish within the field of zeolite synthesis.
