# Supplementary material for: Illuminating the Black Box: A Perspective on Zeolite Crystallization in Inorganic Media
Source: Acc Chem Res. 2023 Aug 11;56(18):2391–402. doi: 10.1021/acs.accounts.3c00269 (PMC10515482; doi:10.1021/acs.accounts.3c00269)
Supplement: Supplementary file 1 — ar3c00269_si_001.pdf [file ar3c00269_si_001.pdf]

Supporting information to:

Illuminating the black box: a perspective on zeolite crystallization in inorganic media.

Karel Asselman[1], Christine Kirschhock[1], Eric Breynaert[1,2*]

Affiliation:

[1] Center for Surface Chemistry and Catalysis – Characterization and Application Team (COK-KAT), KU Leuven, 3001 Leuven, Belgium

[2] NMR-Xray platform for Convergence Research (NMRCoRe), KU Leuven, 3001 Leuven, Belgium

*Corresponding author: eric.breynaert@kuleuven.be

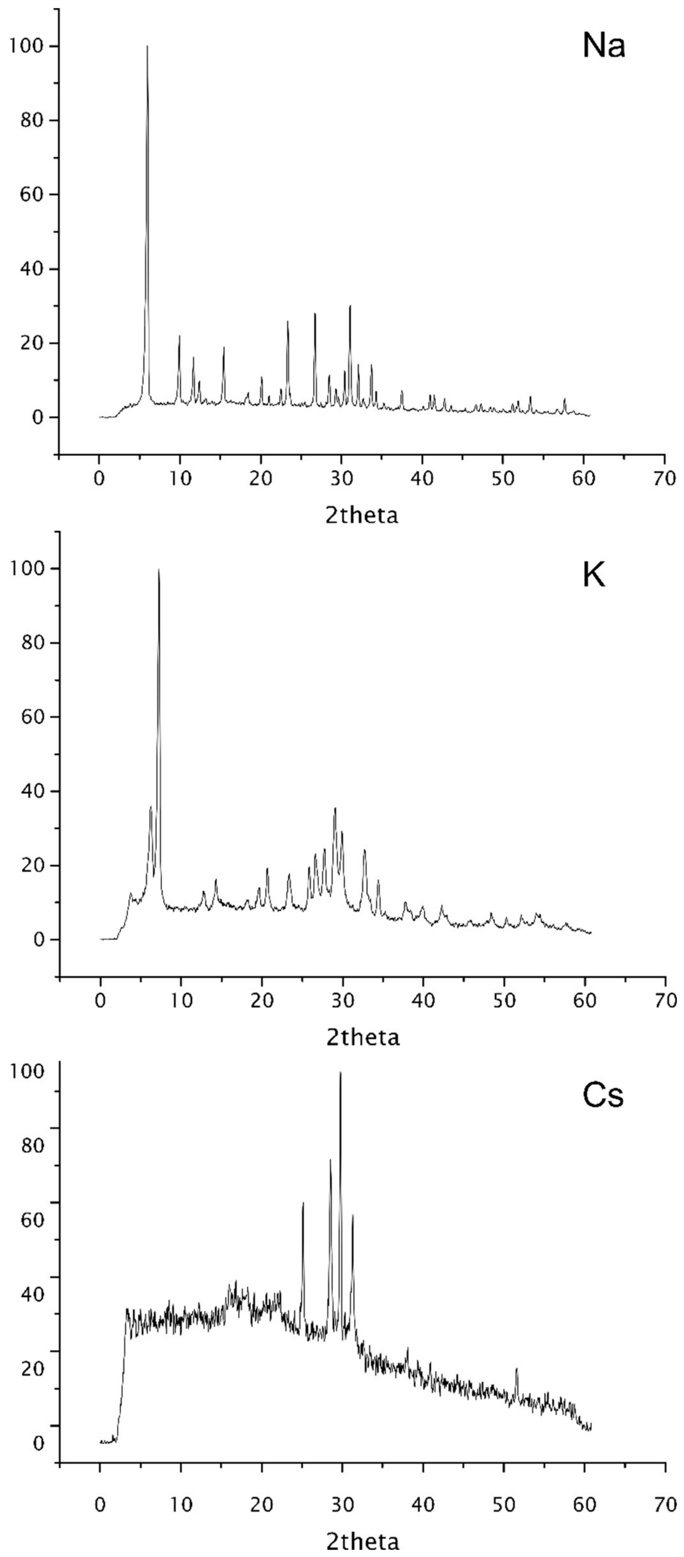

Figure S1: XRD patterns of zeolite phases crystallized from HSIL media at room temperature after 6 months incubation at stationary conditions. Batch stoichiometries were 0.5 SiO_2 – 0.013 Al_2O_3 – 5 MOH – 50 H_2O. Identified topologies for the respective cations (Na, K, Cs) are FAU, BPH and EDI.
